# Assessing Preceptor and Student Perceptions of Remote Advanced Pharmacy Practice Experience Learning during the COVID-19 Pandemic

**DOI:** 10.3390/pharmacy10050103

**Published:** 2022-08-25

**Authors:** S. Lena Kang-Birken, Elaine J. Law, Yong S. K. Moon, Audrey J. Lee, Andrew L. Haydon, Allen Shek

**Affiliations:** Department of Pharmacy Practice, Thomas J. Long School of Pharmacy, University of the Pacific, 3601 Pacific Avenue, Stockton, CA 95211, USA

**Keywords:** pharmacy experiential learning, remote learning, COVID-19, preceptor perception

## Abstract

The coronavirus disease 2019 pandemic created a major shift in learning modalities in the Advanced Pharmacy Practice Experience program. This descriptive study aimed to evaluate preceptor and student perceptions of remote learning experiences and student practice readiness upon completion of remote rotations. Preceptors and students who participated in partial to full remote experiential rotations between 17 August 2020 and 26 March 2021 were invited to complete an on-line survey. A cross-sectional survey consisted of closed-ended questions using a 5-point Likert scale assessing perception on adaptability, effectiveness of remote learning in advancing practice knowledge and skills, and confidence in students’ practice readiness. A total of 29 preceptors and 43 students completed the survey (response rates of 67% and 57%, respectively). Approximately 70% of the remote rotations were practice-based, with ambulatory care representing the most frequently reported rotation by preceptors (38%) and students (28%). A high level of confidence in preceptor perception of their ability to adapt and provide effective remote experiences (average 4.28) matched with the students’ high level of confidence with their preceptors’ abilities (86% agree or strongly agree). Upon the completion of remote rotations, both preceptors and students felt confident in student practice readiness based on student ability to design and initiate individualized patient care plans or complete projects using evidence-based resources (79% and 86%, respectively). Most preceptors (69%) reported that students achieved the rotation objectives at the same level as students engaged in-person experiences. The limitations of remote learning included the absence of direct interactions. Overall, both preceptors and students reported achieving practice readiness with remote experiential learning experiences and felt the remote activities should be continued post-pandemic.

## 1. Introduction

During the coronavirus disease 2019 (COVID-19) pandemic, modifications in workflow and restricted number of personnel including students at the healthcare systems led to the cancellation or restructuring of many Advanced Pharmacy Practice Experience (APPE) Rotations. The Thomas J. Long School of Pharmacy at the University of the Pacific (UOP) is a large pharmacy school located in Northern California with an average class size of 200 students. The APPE curriculum comprises six 6-week APPE rotations from August to May. At the height of the pandemic, the School of Pharmacy (SOP) lost approximately 220 APPE rotations during the last two APPE blocks of the academic year. Many students were quickly transitioned to remote experiential learning or shifted to alternative on-site rotations. During the summer months of 2020, a COVID-19 experiential education task force was created to develop resources for preceptors and students. Remote learning playbooks focusing on four core APPE rotations (ambulatory care, community practice, hospital practice, and internal medicine) were created to provide students and preceptors access to remote APPE activities, sample calendars, and other innovative resources that could be utilized during a full six-week rotation if a student or preceptor were not able to report to the practice site [1]. As the pandemic continued, many students were transitioned to partial or fully remote experience for core or elective APPE rotations.

There are several published research reports describing student feedback and satisfaction following transition from in-person to remote APPEs [2,3]. One study found that students expressed a high percent of strong agreement related to availability of resources and facilitation of autonomous learning during full-time virtual APPEs [2]. However, 16% of students expressed disagreement that virtual learning would help them become better members of a healthcare team. Another study by Singh, H et al. reported outcomes from an asynchronous APPE experience facilitated by faculty, preceptors, and administrators for delivering acute care, ambulatory care, and drug information activities [3]. At the end of their remote APPE, it was concluded that the students achieved acceptable outcomes in evaluating and applying evidence-based medicine in the patient decision-making process and the management of patient-specific goals. 

Much of the literature on remote APPEs and virtual learning during COVID-19 focuses on the student perspective concerning the implementation of remote experiential rotations. In comparison, a paucity of studies have evaluated preceptor perspectives on the impact of COVID-19 on APPEs and effectiveness of remote learning. This report describes preceptor and student perspectives of student practice readiness and satisfaction level after implementation of remote learning activities.

## 2. Materials and Methods

### 2.1. Study Design

Cross sectional surveys of APPE preceptors and students were conducted. A master list of all students who were unable to be on-site between 17 August 2020, and 26 March 2021, was used to identify students who took part in remote APPE activities for four or more days during a six-week APPE rotation. The minimum number of four days was selected based on the School’s APPE policies and procedures which permits up to three days of excused absences per rotation. Based on the selected students who met this criterion, the students’ corresponding APPE preceptors were identified as eligible survey recipients. Recruitment emails containing a direct link to the questionnaire using SurveyMonkey^®^ (San Mateo, CA, USA) were sent to eligible students and preceptors in early-summer 2021 after completion of APPE rotations. The survey was distributed after the students’ graduation to facilitate student feedback without bias or fear of negative feedback from preceptors. Survey participation was voluntary, and the participants were not offered financial or any other incentives to complete the survey. Submissions were anonymous and remained open through the summer semester. The survey research study was approved by the Institutional Review Board at the UOP.

### 2.2. Data Collection

The student survey consisted of 12 questions encompassing three broad categories: demographics, effectiveness of remote learning, and future recommendations post pandemic (Appendix A). There were four background questions on the type of remote APPE rotations, percentage of rotation time conducted remotely, and availability of the electronic health record (EHR) either on-site or remotely. Students were also asked to select types of remote activities they participated in from the following: topic discussions, journal club, patient case presentations, formal presentations, medical team/multi-disciplinary team rounds, telehealth visits, American Society of Health-System Pharmacists competency modules, and other activities. Six closed-ended questions were asked using a 5-point Likert scale (1 = strongly disagree, 2 = disagree, 3 = neither agree nor disagree, 4 = agree, 5 = strongly agree) to assess student perception on effectiveness of remote activities and overall satisfaction. Specific questions included: “My remote activities were effective and advanced my practice knowledge and skills; I feel confident that I can design and initiate individualized patient care plans or projects using evidence-based resources; I feel confident that I can communicate effective care plans, presentations, or projects; My preceptor provided an effective remote learning experience for me; Overall, I was satisfied with the remote activities that APPE rotation offered; I would recommend this remote experience in the future.” Using the same activity list as previously described, students were asked to select specific remote activities they would recommend continuing in the future. For the final open-ended question, students were asked to provide any gaps in the remote experiences.

The preceptor survey consisted of 14 questions (Appendix B). Preceptors were asked to select their practice area and years of practice to describe their background. Additional questions focused on the rotations including the type of remote activities that they assigned students, and the availability of student access to the EHR. There were eight closed-ended questions designed to assess preceptor perception of the students’ adaptability and practice readiness as well as effectiveness of their role as a preceptor using the same 5-point Likert scale. As in the student survey, the preceptors were asked to select the remote activities they planned to continue post-COVID-19. The final open-ended question invited the preceptors to provide their overall reflections about remote experiential learning.

### 2.3. Data Analysis

Preceptor demographics and APPE rotation characteristics were collected and sum-marized. The rotations were categorized as practice-based or project-based when analyz-ing the survey results. Core rotations were all categorized as practice-based rotation. De-pending on the nature of the rotation, elective rotations were classified as either a practice- or project-based rotation. For instance, the geriatric elective rotation was considered as practice-based whereas the managed care elective rotation was considered as project-based. Descriptive analysis of the responses on effectiveness of remote activities concern-ing student practice readiness, confidence and ability to design and initiate individualized patient care plans or projects, and communication skills, was performed. The secondary analysis included perception of preceptor’s ability to deliver remote APPE and their will-ingness to continue to utilize specific remote learning activities post-pandemic.

## 3. Results

### 3.1. Rotation Characteristics

***Student survey.*** Of the 75 eligible APPE students who took part in remote APPE activities and were sent the recruitment email, 43 completed the survey (57% response rate). Most students (81%) participated in remote APPE experiences for 18 days or more during a six-week rotation block (Table 1). More students engaged in remote experiences during practice-based rotations (72%) than project-based rotations (26%). Overall, ambulatory care was the most frequently reported APPE rotation to include remote activities (28%), followed by hospital practice rotation (14%). Among elective rotations, approximately half of the students engaged in project-based rotations in the managed care setting. A diverse range of practice-based elective rotations were reported including geriatrics and critical care. Overall, approximately 61% of students (26/43) had access to EHR among whom most (24/26) were granted remote access to EHR. Students participated in a variety of remote activities with topic discussions (20%), case presentations (20%), formal presentations (17%), and journal club (14%) representing the most reported types.

***Preceptor survey.*** Forty-three preceptors met the inclusion criteria, among whom 29 completed the survey (67% response rate). Most preceptors were experienced practitioners with approximately 76% reporting having at least six years of practice experience and 31% having practiced beyond 15 years (Table 1). Approximately 72% of responders precepted practice-based rotations, primarily ambulatory care rotation (38%). Among project-based elective rotations, pharmaceutical industry (3/8) and managed care rotations (2/8) were more frequently reported. Preceptors reported 72% of students had access to EHR, two-thirds of whom were granted remote access. Of note, remote access to EHR was more readily available to those students on practice-based rotations than project-based rotations (71% vs. 29%, respectively). Similar types and frequencies of remote activities were reported by preceptors as the students: topic discussions (24%), case presentations (20%), formal presentations (18%), and journal club (15%).

### 3.2. Practice Readiness

***Student survey.*** Overall, 84% of students agreed or strongly agreed that their remote learning experiences were effective and advanced their knowledge and skills (Table 2). In a sub-analysis of rotation types, average scores were greater than 4.0 across all rotations except for hospital practice rotation (average score of 3.83). Most students (86%) reported confidence in designing and initiating individualized patient care plans. Of note, a lower level of confidence was reported by students in remote ambulatory care rotations (3.75). Most students felt confident in designing patient care plans or conducting evidence-based projects after completing practice-based rotations or project-based rotations (average scores of 4.03 and 4.36, respectively). Over 90% of students reported confidence in their ability to communicate effectively, especially among those students who completed project-based rotations with an average score of 4.64.

***Preceptor survey.*** As shown in Table 3, 79% of preceptors reported confidence in their student’s ability to design patient care plans and conducting projects (average score of 4.10). For remote ambulatory care rotations, preceptors reported they agreed or strongly agreed that students were able to design drug regimens or projects (4.54) Notably, most preceptors (69%) also reported that students were able to achieve the rotation objectives as the same level as students engaged in-person experiences. However, preceptor perception of student ability to meet rotation objectives was more favorable for project-based rotations than practice-based rotations (average scores of 4.13 and 3.62, respectively). 

### 3.3. Overall Perceptions and Recommendations for the Future

***Student perspective.*** Students were largely satisfied (86%) with their remote activities with an average score of 4.21 (Table 2). Those students engaged in project-based rotations appeared to be more satisfied compared to students in practice-based rotations with average scores of 4.72 and 4.03, respectively. Nearly all students indicated that their preceptors provided an effective remote learning experience (average score of 4.42). Overall, 70% of students reported that they would recommend remote experiences in the future. In a sub-analysis of rotation types, students who completed project-based rotations were more likely to recommend the remote experience in the future compared to those students in practice-based rotations (average scores of 4.64 and 3.69, respectively) which aligned with the students’ overall satisfaction with their remote activities. Notably, the hospital ractice APPE was least recommended to be continuedas a remote experience with an average score of 3.33. Finally, several virtual learning activities were recommended to be incorporated in future rotations with patient case presentations (74%), topic discussions (70%), journal club (58%), formal presentations (58%), and telehealth visits (40%) constituting the most reported activities.

***Preceptor perspective***. Both preceptors and students appeared to demonstrate adaptability to changes in the learning environment. As shown in Table 3, nearly all preceptors reported that students adapted readily to remote learning. Overall, 86% of preceptors reported having access to the necessary technology and felt comfortable utilizing technology to deliver remote learning experiences with an average score of 4.28 (Table 3). Additionally, many preceptors reported receiving support from the SOP with an average score of 3.86. However, approximately a quarter of the preceptors neither agreed nor disagreed, suggesting an opportunity for improvement. Most preceptors (86%) reported that they plan to utilize some of the remote teaching innovations in the future post-pandemic for both project-based rotations and practice-based rotations with average scores of 4.12 and 4.14, respectively. Of note, ambulatory care rotation preceptors were most likely to continue utilizing remote teaching innovations with an average score of 4.64. More specifically, 67% (8/12) of students and 64% (7/11) of preceptors recommended continuing telehealth visits in the future. Finally, preceptors selected similar activities as the students with topic discussions (72%), patient case presentations (66%), journal club (62%), formal presentations (52%), and telehealth visits (38%) representing the most reported activities. 

### 3.4. Free-Response Questions

Feedback from both preceptors and students in free-response questions gave further insight into remote learning experiences (Table 4). Some preceptors commented that remote learning experiences offered valuable opportunities for students and preceptors from other rotations and practice sites to collaborate and engage in topic discussion. In addition, while most preceptors and students agreed that remote learning activities provided meaningful experiences, both groups reported that the absence of direct interdisciplinary team interactions and patient care were missed opportunities and represented a gap in the virtual experiential education.

## 4. Discussion

As the COVID-19 pandemic progressed, many students on APPE rotations were restricted from direct patient care or completely displaced from their rotation sites due to COVID-19 precautions. Since APPE training is part of the graduation and licensure requirements, schools of pharmacy created alternative approaches for completing APPE rotations to facilitate on-time graduation. In the interim, the Accreditation Council for Pharmacy Education provided guidance for APPE rotations and encouraged creative and innovative learning activities if the standards were followed [4]. As such, many schools of pharmacy as well as other healthcare disciplines developed a variety of virtual experiential learning modalities [2,3,5,6]. Activities included transitioning to full-time virtual learning consisting of topic and case discussion, quizzes, and written assignments. To enhance real-life patient care experience, telehealth participation and online resources such as educational electronic medical record or case simulations were utilized [2,5].

During the height of the pandemic, we developed virtual APPE playbooks to provide resources for our students and preceptors in case of partial or full displacement from rotation sites [1]. These playbooks for core rotations sharedseveral learning activities described by other programs, and allowed on-time graduation for our students. In addition, we leveraged our decentralized experiential education model consisting of 17 regions throughout California, each overseen by a faculty regional coordinator with clinical expertise to engage in collaborative precepting between the regions. Such innovative strategies resulted in positive comments from students and preceptors at the time, which led us to conduct surveys to garner formal feedback. There are several published reports describing student feedback and satisfaction following transition to virtual learning [2,3,7,8]. Overall, most students surveyed reported positive learning experiences and indicated potential value in the virtual rotation. However, other studies report common challenges of virtual experiential learning as described in our study. An aggregated sample of weekly reflections from Australian students indicated that virtual education was associated with lack of personal connections, casual interactions, and motivation for engagement in purposeful learning [7]. A cross-sectional survey from a pharmacy school in Canada studied the impact of COVID-19 on students’ personal and professional learning and adaptability. A total of 53 students commented on lack of engagement such as spontaneous discussions with peers and instructors in addition to lack of hands-on learning opportunities [8]. While students’ perspectives on tvirtual experiential learning has been studied, publications on the preceptors’ perspective on effectiveness of remote learning are lacking.

In this descriptive report, our survey results indicated both receptors and students agreed that remote experiential learning was effective. Approximately 70% of preceptors teaching remote rotations reported that their students achieved their rotation objectives at the same level as previous students who took their rotation in-person before COVID-19. These positive findings may be due to the preceptors’ level of experience as 76% of preceptors had six or more years of practice and precepting, allowing them to adapt quickly with changing demands in responsibilities during the pandemic. Thus, many students rated their preceptors favorably in providing an effective learning experience and indicated that remote activities advanced their knowledge and skills. Specifically, 86% of students reported a high level of confidence in designing individualized patient care plans or completing projects using evidence-based resources and 79% of preceptors shared a similar perspective. Interestingly, the preceptors appeared to be more confidence in their students’ ability to design drug regimens or complete evidence-based projects during remote ambulatory care rotations compared to the students’ perspective (average scores of 4.54 and 3.75, respectively).

Most preceptors agreed they planned to incorporate remote teaching innovations such as virtual patient case or topic discussions and formal presentations post-COVID-19, and many students agreed. However, there were some suggestions regarding specific types of APPE rotations by both preceptors and students. There was a high level of satisfaction in achieving rotation objectives and practice readiness from remote learning during project-based rotations, such as the managed care elective, with both preceptors and students recommending this rotation in the future. However, preceptors and students reported a lower level of satisfaction after remote learning activities during practice-based rotations, especially for the hospital pharmacy practice rotation. While students expressed that they had a positive learning experience from virtual APPE playbooks and on-line resources, lack of hands-on experience was cited as a significant challenge for them as similarly reported by medical students participating in a virtual orthopedic inpatient clinical clerkship [6]. Since virtual experiences cannot replace direct patient care or hands-on pharmacy operational experiences in an inpatient setting, we encouraged our inpatient preceptors to transition back to in-person experiential education when authorized at their institution. However, in ambulatory care settings telehealth was quickly adopted. The Centers for Disease Control and Prevention reported 154% increase in telehealth visits since the emergence of the COVID-19 pandemic in late March 2020 [9]. Among 2000 patients who received a minimum of one telehealth visit, 79% of patients were satisfied with their telehealth visits [10]. Opportunities to participate in patient interaction and care using telehealth during ambulatory care rotations is likely to have contributed to favorable responses by both preceptors and students in our survey.

While the survey was conducted after the students’ graduation to promote their non-biased feedback, our study has a few limitations due to the nature of a volunteer survey study design.. In addition to a modest surveyresponse rate, preceptor perception of virtual learning experiences the rotation could not be directly compared to that of the corresponding student. There were also unequal numbers of practice-based and project-based rotations making it difficult to evaluate the overall experiences. Finally, a high portion of students who were granted access to EHR may have affected the overall experience more positively.

## 5. Conclusions

This descriptive report uniquely describes preceptor and student perceptions of APPE rotations utilizing remote activities during COVID-19. Although students lacked in-person interactions with patients and other health care providers, both preceptors and students reported that remote experiential learning experiences were effective. Overall, preceptors reported that students engaged in remote experiences achieved a similar level of practice readiness as students participating in on-site rotations. Both preceptors and students suggested integration of remote activities into APPE rotations post-pandemic.

## Figures and Tables

**Table 1:** APPE Student and Preceptor Surveys: Preceptor demographics and rotation characteristics.

	APPE Student Survey (n= 43) (%)	APPE Preceptor Survey (n = 29) (%)
**Preceptor years of practice**		
1–5 years	NA	7 (24.1%)
6–10 years	NA	9 (31.0%)
11–15 years	NA	4 (13.8%)
Greater than 15 years	NA	9 (31.0%)
**Percent of APPE rotation conducted remotely**		
81–100% (between 24 and 30 days)	33 (76.7%)	NA
61–80% (between 18 and 24 days)	2 (4.7%)	NA
41–60% (between 12 and 18 days)	5 (11.6 %)	NA
21–40% (between 6 and 12 days)	2 (4.7 %)	NA
Less or equal to 20% (6 days or less)	1 (2.2 %)	NA
**Type of APPE rotation with remote experiences**		
**Practice-based Core rotations**	20/43 (46.2%)	16/29 (55.2%)
Ambulatory care	12 (27.9%)	11 (37.9%)
Hospital practice	6 (13.6%)	2 (6.9%)
Internal medicine	2 (4.7%)	2 (6.9%)
Community practice	NA	1 (3.5%)
**Elective rotations**	23/43 (53.5 %)	13/29 (45.0%)
**Project-based electives**	12/43 (27.9%)	8/29 (28%)
Managed care	8 (18.6%)	2 (6.9%)
Pharmaceutical industry	1 (2.3%)	3(10.3%)
Other ^a,b^	3 (7.0%) ^a^	3 (10.3%) ^b^
**Practice-based electives**	11/43 (25.6%)	5/29 (17.2%)
Geriatrics (Long-term care)	3 (6.9%)	1 (3.4%)
Critical care	2 (4.7%)	1 (3.4%)
Others ^c,d^	6 (14.0%) ^c^	3 (10.3%) ^d^
**Types of remote experiences (Total number)**	133	102
Patient case presentations	27 (20.3%)	20 (19.6%)
Topic discussions	26 (19.5%)	24 (23.5%)
Formal presentations	23 (17.3%)	18 (17.6%)
Journal club	18 (13.5%)	15 (14.7%)
Telehealth visits	14 (10.5%)	8 (7.8%)
Multi-Disciplinary team rounds	8 (6.0%)	6 (5.9%)
ASHP competency modules	6 (4.5%)	3 (2.9%)
Virtual clean room	2 (1.5%)	NA
Other ^e,f^	9 (6.8%) ^e^	8 (7.8%) ^f^

NA = Not Applicable; ASHP = American Society of Health System Pharmacists; IT = Information Technology; ^a^ Other project-based electives from student survey: Drug information 1 (2.3%), Medication safety 1 (2.3%), and Pharmacy and Therapeutics 1 (2.3%); ^b^ Other project-based elective from preceptor survey: Academia 1 (3.4%), Administration 1 (3.4%), and Medication safety 1 (3.4%); ^c^ Other practice-based electives from student survey: Infectious Disease 1 (2.3%), Advanced Ambulatory Care 1 (2.3%), Diabetes Management 1 (2.3%), Oncology 1 (2.3%), Psychiatry 1 (2.3%), and Specialized Pharmacy 1 (2.3%); ^d^ Other Practice-Based Electives from Preceptor Survey: Infectious Diseases 1 (3.4%), Oncology 1 (3.4%), and Specialized Pharmacy 1 (3.4%); ^e^ From Student Survey: Attending meetings, attending lectures, developing policy and procedures, filming education videos on use of a glucometer, insulin, and drug pen, completing a research project and presentation, answering pharmacist drug-information queries, completing a drug monograph, updating protocols or completing other projects assigned by preceptor; ^f^ From Preceptor Survey: Attending meetings, preparing, and presenting drug Information and monograph presentations, performing medication safety utilization review audits, completing adverse event and medication error reports, performing project-based research activities, completing clinical trial management projects, participating in e-learnings, acquiring hands-on IT experience in a “sandbox environment”, creating digital materials, conducting patient interviews, and engaging in SOAP presentations and discussions.

**Table 2:** Student survey results (N = 43).

Questions	Weighted Average (out of 5)	% Agree or Strongly Agree
My remote activities were effective and advanced my practice knowledge and skills	4.16	83.7%
I feel confident that I can design and initiate individualized patient care plans or projects using evidence-based resources	4.12	86%
I feel confident that I can communicate effective care plans, presentations, or projects (verbal or written)	4.30	93%
My preceptor provided an effective remote learning experience for me	4.42	90.7%
Overall, I was satisfied with the remote activities that this APPE rotation offered	4.21	86%
I would recommend this remote experience in the future	3.93	69.8%

**Table 3:** Preceptor survey results (N = 29).

Questions	Weighted Average (out of 5)	% Agree or Strongly Agree
Students were able to adapt readily to the remote APPE learning environment	4.38	89.7%
Students achieved the rotation objectives at the same level as in-person	3.76	68.9%
I felt confident in my ability to deliver an effective remote APPE rotation	4.28	86.2%
I feel confident that students in a remote APPE can design and initiate individualized patient care plans or projects using evidence-based resources	4.10	79.3%
I received support from School(s) of Pharmacy to help manage my remote APPE rotation(s)	3.86	68.9%
I had access to the necessary technology platforms and reliable internet to deliver an effective remote APPE rotation	4.28	86.2%
I felt comfortable with the technology available to deliver an effective remote APPE rotation	4.28	86.2%
I plan to continue utilizing some remote teaching innovations in the future post COVID-19	4.14	86.2%

APPE: Advanced Pharmacy Practice Experience; COVID-19 Coronavirus disease 2019.

**Table 4:** Preceptor and student free-response questions.

Preceptor: Additional comments not addressed in the survey
“Remote interactions for case and topic presentations were conducted with interns at other sites”
“I appreciate all the resources and help from the Regional Coordinator to try and make the rotations work well and have proper content.”
“I just feel like the in-person connection was lost during those times but learning and work continued.”
“Remote rotations are extremely difficult for students in the critical care/internal medicine rotations especially if they have no hospital experience.”
“I run to codes and traumas, and they are not able to experience what they would be able to experience if they were on campus. I strongly do not recommend this rotation remotely.”
“While it served the purpose during quarantine, having the right student is key to virtual learning.”
“Public speaking skills cannot fully be practiced remotely. Body language, preparation, and overcoming nervousness is not the same level when one can read off a document at home vs speaking in front of a large live audience.”
“Some of the research for med system issues is best done in person. It was fine to work around this for COVID, but ideally the learning experience is best at least partially or fully on-site.”
“This elective industry rotation was completely project-based. I am now fully remote and plan to continue my APPE rotations as fully/partially remote moving forward.
**Student: What gaps remained in the remote rotation experiences?**
“Interdisciplinary care, direct patient care”
“It is difficult to build connections with preceptors in a virtual setting and can be difficult to keep the audience’s attention during presentation”
“Missing out on some of the inpatient experiences in the main pharmacy to see how it operated. Another would be missing rounds due to being remote”
“Unable to conduct comprehensive care and to do hands on activities that in-clinic appointments provided”
“The dynamic was very different. It (critical care rotation) started online, then became onsite. There were more opportunities to learn on site when things came up. I was able to observe a code blue.”
“Access to the EHR, ability to make in-person connection”
“It would have been nice to see my preceptors in person, but this rotation was a good rotation to do virtually”
“Much rather do remote even on other rotations. It would make me feel a lot safer, and that would make me learn better as well. Honestly there’s not much difference on site vs virtual”
“The biggest gap comes with not having remote access and also keeping up with the work and deadlines simply because it’s so comfortable at home”

## Appendix A. Student Perception Survey of Remote Advanced Pharmacy Practice Experiential Learning

1. APPE rotation conducted remotely (select one):
  ◯ Community Practice
  ◯ Ambulatory Care
  ◯ Internal Medicine
  ◯ Hospital Practice
  ◯ Elective: Please specify
2. Percentage of APPE rotation conducted remotely:
  ◯ Fall (8/2020–12/2020)
    ○ 81–100% (between 24 and 30 days)
    ○ 61–80% (between 18 and 24 days)
    ○ 41–60% (between 12 and 18 days)
    ○ 21–40% (between 6 and 12 days)
    ○ ≤20% (6 days or less)
  ◯ Fall (8/2020–12/2020)
    ○ 81–100% (between 24 and 30 days)
    ○ 61–80% (between 18 and 24 days)
    ○ 41–60% (between 12 and 18 days)
    ○ 21–40% (between 6 and 12 days)
    ○ ≤20% (6 days or less)
3. Did you have access to Electronic Health Record?
  ◯ Yes (select all that apply)
    ▯ Remote access
    ▯ On-site access
  No
4. What type of remote APPE activities did you participate in (select all that apply):
  ◯ Topic discussions
  ◯ Journal club
  ◯ Patient case presentation
  ◯ Formal presentations (ex. P&T committee, in-services, staff meetings, etc)
  ◯ Medical Team /Multi-disciplinary Team Rounds
  ◯ Telehealth visits
  ◯ ASHP competency modules
  ◯ Others, please specify

Please indicate your level of agreement with each of the following statements (0 = n/a, 1 = Strongly Disagree to 5 = Strong Agree):						
	**0** **n/a**	**1** **Strongly Disagree**	**2** **Disagree**	**3** **Neither Agree nor Disagree**	**4** **Agree**	**5** **Strongly Agree**
5. My remote activities were effective and advanced my practice knowledge and skills		**◯**	**◯**	**◯**	**◯**	**◯**
6. I feel confident that I can design and initiate individualized patient care plans or projects using evidence-based resources	**◯**	**◯**	**◯**	**◯**	**◯**	**◯**
7. I feel confident that I can communicate effective care plans, presentations, or projects (verbal or written)	**◯**	**◯**	**◯**	**◯**	**◯**	**◯**
8. Overall, I was satisfied with the remote activities that this APPE rotation offered		**◯**	**◯**	**◯**	**◯**	**◯**
9. I would recommend this remote experience in the future		**◯**	**◯**	**◯**	**◯**	**◯**

10. Which parts of the remote rotation experiences should be continued? (select all that apply)
  ◯ Topic discussions
  ◯ Journal club
  ◯ Patient case presentation
  ◯ Formal presentations (ex. P&T committee, in-services, staff meetings, etc.)
  ◯ Medical Team/Multi-disciplinary rounds
  ◯ Telehealth visits
  ◯ ASHP Competency modules
  ◯ Virtual cleanroom
  ◯ Others, please specify
11. My preceptor provided an effective remote learning experience for me
12. Free text response: What gaps remained in the remote rotation experiences?

## Appendix B. Preceptor Perception Survey of Remote Advanced Pharmacy Practice Experiential Learning

1. Practice Area (select one):
  ◯ Community Practice
  ◯ Ambulatory Care
  ◯ Internal Medicine
  ◯ Hospital Practice
  ◯ Elective: Please specify
2. Years of practice (select one):
  ◯ 1–5 years
  ◯ 6–10 years
  ◯ 11–15 years
  ◯ >15 years
3. Did the students have access to Electronic Health Record?
  ◯ Yes (select all that apply)
    ▯ Remote access
    ▯ On-site access
  No
4. What type of remote APPE activities did you utilize (select all that apply):
  ◯ Topic discussions
  ◯ Journal club
  ◯ Patient case presentation
  ◯ Formal presentations (ex. P&T committee, in-services, staff meetings, etc)
  ◯ Medical Team /Multi-disciplinary Team Rounds
  ◯ Telehealth visits
  ◯ ASHP competency modules
  ◯ Others, please specify

Please indicate your level of agreement with each of the following statements (1 = Strongly Disagree to 5 = Strong Agree):					
	1 Strongly Disagree	2 Disagree	3 Neither Agree nor Disagree	4 Agree	5 Strongly Agree
5. Students were able to adapt readily to the remote APPE learning environment.	**◯**	**◯**	**◯**	**◯**	**◯**
6. I felt confident in my ability to deliver an effective remote APPE rotation.	**◯**	**◯**	**◯**	**◯**	**◯**
7. I received support from School(s) of Pharmacy to help manage my remote APPE rotation(s).	**◯**	**◯**	**◯**	**◯**	**◯**
8. Students achieved the rotation objectives at the same level as in-person.	**◯**	**◯**	**◯**	**◯**	**◯**
9. I feel confident that students in a remote APPE can design and initiate individualized patient care plans or projects using evidence-based resources	**◯**	**◯**	**◯**	**◯**	**◯**
10. I had access to the necessary technology platforms and reliable internet to deliver an effective remote APPE rotation.	**◯**	**◯**	**◯**	**◯**	**◯**
11. I felt comfortable with the technology available to deliver an effective remote APPE rotation.	**◯**	**◯**	**◯**	**◯**	**◯**
12. I plan to continue utilizing some remote teaching innovations in the future post COVID	**◯**	**◯**	**◯**	**◯**	**◯**

13. Which parts of the remote rotation experiences should be continued? (select all that apply)
  ◯ Topic discussions
  ◯ Journal club
  ◯ Patient case presentation
  ◯ Formal presentations (ex. P&T committee, in-services, staff meetings, etc.)
  ◯ Medical Team/Multi-disciplinary rounds
  ◯ Telehealth visits
  ◯ ASHP Competency modules
  ◯ Virtual cleanroom
  ◯ Others, please specify
14. Optional (free text)

Please include any additional comments not addressed in this survey that you wish to share regarding your experiences with remote rotations during COVID.
